# DUAL-Net: Joint Domain-Invariant and User-Adaptive Feature Learning for Gesture Recognition

**DOI:** 10.3390/s26134182

**Published:** 2026-07-02

**Authors:** Shuangjiao Zhai, Bo Yang, Zixin Dai, Yujie Guo, Baojin Jing, Jia Qin, Pinle Qin

**Affiliations:** 1 School of Computer Science and Technology, North University of China, Taiyuan 030051, China; s202307006@st.nuc.edu.cn (Z.D.); s202407021@st.nuc.edu.cn (Y.G.); s202407026@st.nuc.edu.cn (B.J.); jqin@nuc.edu.cn (J.Q.); qpl@nuc.edu.cn (P.Q.); 2 School of Mechanical Engineering, Taiyuan University of Technology, Taiyuan 030600, China

**Keywords:** WiFi CSI, WiFi–vision fusion, gesturerecognition, domain-invariant learning, cross-user adaptation, diffusion generation

## Abstract

Human activity recognition has become an important component of human–computer interaction and ubiquitous computing. Among various sensing technologies, WiFi-based gesture recognition has attracted increasing attention due to its contactless nature and robustness to visual occlusion. However, environmental variations and user-specific differences often lead to significant performance degradation, particularly in cross-user scenarios. Existing methods primarily focus on learning domain-invariant representations, which may overlook user-specific characteristics that are essential for accurate recognition. To address this issue, we propose the Domain-invariant and User-Adaptive Learning Network (DUAL-Net), a dual-branch framework that jointly models domain-invariant and user-adaptive representations. Specifically, DUAL-Net incorporates a contrastive fusion learning (CFL) module with modality-specific encoders to learn complementary representations from WiFi and vision modalities. Furthermore, a spatial matrix difference (SMD)-guided cross-modal generation (CMG) module is introduced to generate user-adaptive WiFi features by incorporating structural priors derived from skeletal representations. To improve deployment efficiency, DUAL-Net adopts a two-stage learning framework, where adaptation is conducted offline to reduce online computational overhead. Experiments on the MM-Fi dataset and a self-collected dataset show that DUAL-Net achieves superior cross-user recognition performance compared with existing single-modality and multimodal methods. In addition, SMD-guided conditioning improves recognition accuracy by up to 8.79% over diffusion generation without structural guidance.

## 1. Introduction

Human activity recognition (HAR) is vital for intelligent interaction in fields such as smart homes [1,2,3], healthcare [4], and autonomous driving [5]. Accurate behavior recognition significantly enhances user experience, system efficiency, and safety. Recent advances have explored multiple sensing modalities, notably vision-based, sensor-based, and WiFi-based approaches [6,7,8,9]. Vision-based techniques excel in capturing fine-grained spatial information but remain vulnerable to illumination changes, occlusions, and viewpoint limitations. Sensor-based methods leveraging accelerometers and gyroscopes reliably record motion data but require users to wear devices, which reduces practicality and comfort. In contrast, WiFi-based HAR has attracted increasing attention for its contactless sensing, affordability, and resilience to visual constraints. However, WiFi-based HAR approaches are still highly susceptible to environmental interference, such as background variations and user location [10]. Moreover, existing WiFi-based HAR methods typically emphasize domain-invariant features to improve robustness, yet they often overlook user-dependent variations in gesture execution style, speed, and amplitude. This omission limits their ability to generalize in real-world applications [11].

Motivated by these limitations, recent studies have investigated domain adaptation techniques and user-independent approaches, aiming to extract stable features across diverse environments and generalize across users [12,13,14]. However, these two lines of work typically address domain variations and user differences in isolation, overlooking the intricate interplay between environmental conditions and individual behavioral patterns. In addition, WiFi signals inherently lack the spatial resolution required to capture fine-grained, user-specific gesture details, which further constrains recognition performance in practical scenarios.

To address these limitations, multimodal learning has emerged as a promising direction that combines complementary sensing modalities to enhance feature robustness [15]. For instance, it has been successfully applied in domains such as audio–visual sentiment analysis [16], vision–language navigation [17], and WiFi–vision-based gait recognition [18]. In particular, WiFi–vision-based gesture recognition integrates the high-resolution spatial sensing of vision sensors with the robustness of WiFi signals against occlusion and illumination changes, effectively overcoming key limitations of single-modality systems [19,20]. However, WiFi–vision fusion still faces critical challenges similar to those in broader multimodal learning. In particular, structural and temporal mismatches between modalities, along with incomplete or missing data in real-world deployments, often lead to unstable fusion and degraded recognition performance [21,22]. These limitations are further compounded by the need to adapt to unseen users, where collecting personalized WiFi data is costly and impractical at scale.

**Figure 1:**
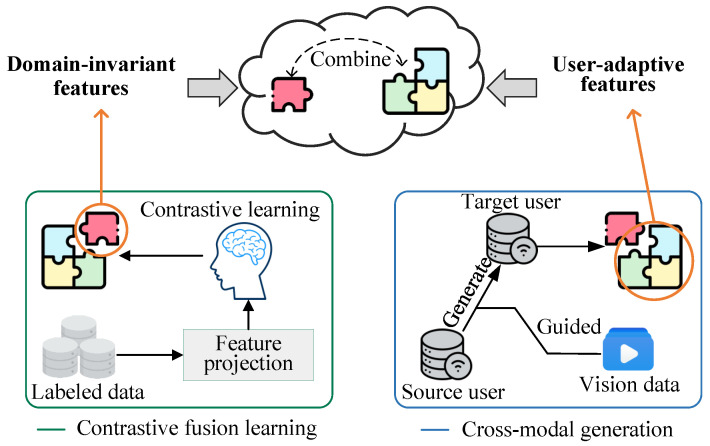
The workflow of DUAL-Net. Existing methods emphasize domain-invariant features, while DUAL-Net additionally learns user-adaptive features to capture individual differences.

To address these challenges, we propose the Domain-invariant and User-Adaptive Learning Network (DUAL-Net), a dual-branch framework that jointly models domain-invariant and user-adaptive representations. As illustrated in Figure 1, existing methods primarily emphasize domain-invariant representations, which may overlook user-specific characteristics that are essential for accurate recognition. DUAL-Net addresses this limitation by complementing domain-invariant representations with user-adaptive representations, enabling the model to capture both shared gesture semantics and individual user characteristics.

The first branch is a contrastive fusion learning (CFL) module designed to learn domain-invariant representations. Rather than directly enforcing cross-modal alignment, CFL employs modality-specific encoders to extract complementary information from WiFi and vision modalities and projects them into a shared semantic space. Contrastive learning is then applied to encourage representations of the same gesture class to remain close while separating those of different classes. This process enables the model to learn stable gesture semantics while reducing the influence of environmental variations.

However, domain-invariant representations alone are insufficient for modeling user-specific differences. To address this issue, DUAL-Net introduces a spatial matrix difference (SMD)-guided cross-modal generation (CMG) module. Specifically, skeletal keypoints extracted from vision data are transformed into spatial matrices to characterize structural differences between source and target users. The resulting SMD quantifies user-specific discrepancies and is incorporated as a conditioning signal to guide diffusion-based feature generation. By introducing structural priors into the generation process, CMG synthesizes user-adaptive WiFi representations for unseen users without requiring additional WiFi data collection. Finally, the domain-invariant and user-adaptive representations are fused for gesture recognition.

To improve deployment efficiency, DUAL-Net adopts a two-stage learning framework. The CFL and CMG modules are performed during offline learning, while online inference avoids repeated multimodal adaptation and diffusion-based feature generation, thereby reducing computational overhead. We evaluate the proposed DUAL-Net framework on two datasets, namely the publicly available MM-Fi dataset and a self-collected multimodal gesture dataset. Experimental results demonstrate that DUAL-Net significantly outperforms single-modality baselines by 24.7% in recognition accuracy and surpasses existing multimodal approaches by 14.5%. Furthermore, it exhibits strong generalization, achieving over 90% accuracy in both cross-user and cross-domain scenarios.

The key contributions of this paper are summarized as follows:

- We propose DUAL-Net, a dual-branch WiFi–vision gesture recognition framework that jointly models domain-invariant and user-adaptive representations, addressing the limitations of domain-invariant feature learning in cross-user scenarios.
- We design a contrastive fusion learning (CFL) module with modality-specific encoders, which learns domain-invariant representations from WiFi and vision modalities while avoiding direct cross-modal alignment, producing stable and complementary gesture semantics.
- We introduce a spatial matrix difference (SMD)-guided cross-modal generation (CMG) module, which encodes skeletal information into spatial matrices to characterize structural differences across users and uses them as conditional signals for diffusion-based generation, enabling user-adaptive WiFi feature synthesis for unseen users.

## 2. Related Work

Robust gesture recognition has been widely studied from different perspectives. In this paper, we review related work along three major lines of research. The first concerns WiFi-based gesture recognition, which exploits wireless signals for contactless activity sensing. The second addresses domain-invariant and user-adaptive recognition, which aims to improve generalization across diverse environments and users. The third focuses on multimodal learning, with particular attention to WiFi–vision fusion that integrates complementary sensing modalities. These three research directions form the basis of our proposed DUAL-Net framework.

### 2.1. WiFi-Based Gesture Recognition

With the widespread deployment of wireless infrastructure, WiFi signals have evolved beyond their role as communication carriers to serve as key enablers of wireless sensing [23,24]. In the context of gesture recognition, human movements influence the multipath propagation of WiFi signals, introducing variations in the phase and amplitude of Channel State Information (CSI). These perturbations encode motion-related patterns that can be leveraged to recognize user gestures in a contactless and device-free manner.

Early WiFi-based gesture recognition systems primarily relied on handcrafted time and frequency domain features such as amplitude extrema, mean, standard deviation, signal energy, and frequency peaks [25,26,27,28]. These features were typically classified using models such as Support Vector Machines (SVMs), k-Nearest Neighbors (KNNs), or Random Forests (RFs), and in some cases matched through dynamic time warping (DTW). While efficient and interpretable, these approaches often struggled to generalize due to fixed feature definitions and rigid decision boundaries. To overcome these limitations, subsequent research adopted deep learning techniques, employing CNNs and LSTMs to automatically capture spatial and temporal dependencies from raw CSI [29,30,31]. Other studies transformed CSI into spectrogram-like representations to exploit image-based architectures [32]. These models achieved higher accuracy and robustness but continued to face challenges when applied to new users or unseen environments. In addition, compared to visual signals, WiFi inherently provides limited semantic richness due to its coarse spatial resolution and indirect encoding of human motion. As a result, it remains difficult to capture subtle gesture variations across different users, which hinders generalization in cross-user and cross-domain scenarios. Related studies on scalable indoor sensing also show that WiFi-based systems remain affected by data quality, user participation, device burden, and environmental dynamics [33,34]. These findings further highlight the need to improve robustness and adaptability in practical WiFi sensing systems.

### 2.2. Domain-Invariant and User-Adaptive Gesture Recognition

Achieving robust gesture recognition across different environments and users remains a fundamental challenge in WiFi-based sensing. Spatial layout variations, signal interference, and user-specific gesture styles often lead to significant shifts in WiFi signal distributions, thereby compromising the generalization ability of recognition models. To address these issues, prior research has primarily explored two directions. One focuses on domain-invariant learning to mitigate environmental variations, while the other emphasizes user-adaptive learning to handle user-specific differences.

Domain-invariant techniques aim to reduce performance degradation caused by environmental changes. These approaches typically align feature distributions between source and target domains through adversarial learning or distance-based regularization. For example, adversarial training frameworks introduce a domain classifier that guides the feature extractor to learn domain-invariant representations [35,36]. Metric-based approaches such as Maximum Mean Discrepancy (MMD) have also been applied to reduce domain discrepancy in the feature space [37,38]. Recent domain generalization studies further indicate that robust representations should not only reduce domain-specific variations but also preserve class-discriminative information [39]. In WiFi-based gesture recognition, methods such as WiAG [40] and DPSense [41] generate virtual samples or apply signal enhancement to improve robustness under dynamic environmental conditions. However, these works usually assume consistent gesture execution styles across different users and often fail to capture user-related variability.

In parallel, user-adaptive approaches seek to suppress irrelevant user-specific traits while retaining gesture semantics. For instance, CrossGR [14] employs a generative adversarial network to disentangle gesture-relevant information from user-dependent characteristics, thereby producing more generalized representations. Other works leverage few-shot learning and meta-learning strategies to improve recognition performance on unseen users using limited training data [42,43]. Although these methods enhance cross-user generalization, they often treat domain shifts and user variability as independent challenges without modeling their interdependence.

In practice, however, domain and user factors are often closely coupled. A user’s gesture style may interact differently with WiFi signal patterns depending on environmental conditions such as room geometry, device orientation, or propagation paths. Recognition models that separately address domain-invariant learning and user-adaptive modeling may fail to capture these intertwined effects. This limitation highlights the need for integrated frameworks that can simultaneously extract stable domain-invariant features and adapt to user-specific variations. The proposed DUAL-Net framework addresses this gap by jointly incorporating domain-invariant and user-adaptive representations within a unified recognition pipeline, thereby improving performance under both cross-domain and cross-user conditions.

### 2.3. Multimodal Learning and WiFi–Vision Fusion

Multimodal learning has emerged as a promising direction for improving recognition performance by integrating complementary information from multiple sensing modalities [44]. In the context of human activity understanding, combining WiFi signals with vision data helps overcome the inherent limitations of each modality. WiFi signals are resilient to lighting changes and occlusions but suffer from low spatial resolution and limited semantic expressiveness, whereas vision signals provide rich spatial detail but are vulnerable to visual disturbances. Fusing these two modalities therefore offers the potential to improve both robustness and accuracy in complex and dynamic environments. However, multimodal adaptation remains challenging because heterogeneous domain shifts, cross-modal dependency preservation, modality imbalance, and missing inputs are difficult to address using unimodal alignment strategies alone [45].

Recent research has explored WiFi–vision fusion for a range of human sensing tasks, including activity recognition [46,47], gait recognition [48], and gesture recognition [20]. Zou et al. [46] employed decision-level fusion of WiFi and RGB video to enhance recognition robustness under degraded visual conditions. However, this strategy captures only limited cross-modal interactions, thereby restricting the benefits of fusion. Guo et al. [47] proposed a feature-level fusion framework that projects WiFi and vision data into a shared latent space to improve action recognition. While effective, this approach may not fully exploit the complementary characteristics of the two modalities. WiVi [48] developed a multi-path gait recognition system that matches WiFi-derived features with a visual gait library, but performance degrades in scenarios with closely spaced walking paths due to interference. More recently, WiVi-GR [20] introduced a gesture recognition framework based on channel superposition convolution and data-level fusion, incorporating comprehensive speed representations to improve classification accuracy. However, effectively modeling inter-modal correlations remains an open challenge. Many existing approaches rely on fixed fusion strategies and struggle with signal heterogeneity or incomplete modality data. These limitations underscore the need for more flexible and adaptive multimodal frameworks that can dynamically align heterogeneous signals and maintain robustness when only partial modality inputs are available.

Although substantial progress has been made in these three research directions, each line of work still faces critical limitations. WiFi-based gesture recognition suffers from environmental sensitivity and limited semantic expressiveness. Domain adaptation and user-independent techniques improve robustness but often treat domain and user variability in isolation, overlooking their complex interplay. Multimodal learning, especially WiFi–vision fusion, mitigates some of these issues but still struggles with modality heterogeneity and incomplete data in practical deployments. These challenges highlight the need for a unified framework that can simultaneously capture stable domain-invariant features and preserve user-adaptive characteristics. DUAL-Net is proposed to address this gap by jointly modeling domain-invariant and user-adaptive representations for robust gesture recognition.

## 3. System Design

This section introduces the proposed DUAL-Net framework, which jointly models domain-invariant and user-adaptive representations within a unified WiFi–vision architecture. An overview of the framework is first provided, followed by detailed descriptions of its three core modules. To learn stable cross-modal representations, the contrastive fusion learning (CFL) module employs modality-specific encoders to extract complementary WiFi and vision features and optimizes a contrastive objective in a shared embedding space. Instead of directly enforcing feature alignment, this design encourages instance-level consistency while preserving modality-specific characteristics. To capture user-specific gesture variations, the spatial matrix difference (SMD)-guided cross-modal generation (CMG) module generates user-adaptive WiFi representations conditioned on skeletal structures extracted from vision data. The proposed SMD encodes structural differences between users and serves as a conditioning signal for diffusion-based generation, enabling adaptation to unseen users without additional WiFi data collection. Finally, the domain-invariant and user-adaptive representations are fused for gesture recognition. The entire framework follows a two-stage learning paradigm, where CFL and CMG are performed during offline training, while online inference is lightweight with reduced computational overhead.

### 3.1. Overview of the DUAL-Net Framework

**Figure 2:**
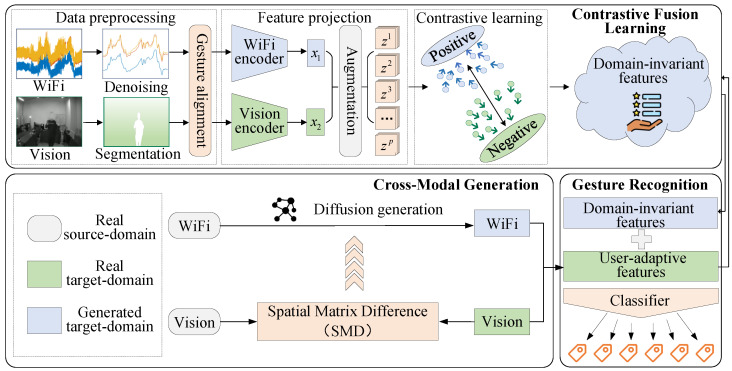
Overview of DUAL-Net. The framework learns domain-invariant features through contrastive WiFi–vision fusion, generates user-adaptive WiFi features via SMD-guided diffusion, and combines both feature types for cross-user and cross-environment gesture recognition.

As shown in Figure 2, the proposed DUAL-Net framework consists of three core modules: a contrastive fusion learning (CFL) module, a cross-modal generation (CMG) module, and a gesture recognition module. The CFL module first processes raw WiFi and vision inputs. WiFi signals are denoised using a Butterworth low-pass filter, while segmentation techniques are applied to vision data to isolate gesture-relevant regions. After temporal alignment, both modalities are encoded by modality-specific encoders and projected into a shared semantic space. Instead of enforcing direct cross-modal alignment, contrastive learning is applied to maximize intra-class similarity and increase inter-class separability, resulting in stable domain-invariant representations. However, domain-invariant features alone are insufficient to capture user-specific characteristics required for cross-user recognition. To address this limitation, the CMG module generates user-adaptive WiFi representations conditioned on visual inputs using a diffusion-based generative model. Specifically, skeletal keypoints are transformed into condition vectors via a spatial matrix difference (SMD) formulation, which provides structural guidance for the diffusion process and enables generation tailored to target users. This design eliminates the need for collecting WiFi data from target users, improving adaptability while reducing deployment cost. Finally, the gesture recognition module fuses domain-invariant and user-adaptive features for classification.

Together, these modules form a unified framework rather than independent components. The overall design jointly learns domain-invariant representations and user-adaptive features to address the limitations of existing WiFi-based gesture recognition methods under environmental variations and inter-user differences. This integrated design improves recognition performance by capturing both shared gesture semantics and user-specific characteristics. In addition, it enables adaptation to unseen users without requiring additional WiFi data collection, thereby reducing data acquisition cost and supporting practical deployment scenarios.

### 3.2. Contrastive Fusion Learning Module

As depicted in Figure 2, the contrastive fusion learning module is composed of three main components, namely data preprocessing, feature projection, and contrastive learning. In the preprocessing stage, WiFi signals are denoised using a Butterworth low-pass filter to suppress high-frequency noise while preserving gesture-relevant temporal structures, and segmentation methods are applied to vision data to isolate gesture-relevant regions. After temporal alignment, the two modalities are encoded by modality-specific encoders and projected into a shared latent space. Finally, contrastive learning is applied to maximize intra-class similarity while minimizing inter-class discrepancy, enabling the model to extract gesture-relevant semantics that remain robust under varying environmental conditions.

#### 3.2.1. Data Preprocessing

WiFi and vision data collected in daily environments are often corrupted by diverse types of noise, leading to degraded feature quality and reduced recognition performance. To address this challenge, a series of preprocessing steps are applied to denoise the input signals, isolate gesture-relevant regions, and ensure temporal alignment across modalities.

For the WiFi modality, Channel State Information (CSI) is extracted from the physical layer, providing fine-grained measurements of amplitude and phase across multiple subcarriers. However, CSI is highly vulnerable to ambient interference, which introduces instability and obscures gesture-related dynamics. To suppress such interference while preserving meaningful variations, a Butterworth filter is applied to the raw CSI sequences.

For the vision modality, RGB video data is first converted to grayscale to reduce computational overhead while retaining essential motion cues. Foreground segmentation is then performed using the $U^{2}$-Net model [49], effectively isolating the subject from background clutter and highlighting gesture-relevant regions.

Finally, to ensure temporal consistency between WiFi and vision streams, a time-series alignment procedure is introduced. In the WiFi data, gesture execution is detected through variance-based thresholding of amplitude fluctuations, marking the onset and offset of each gesture. In parallel, average optical flow magnitudes are computed across video frames, and adaptive thresholds are applied to localize gesture boundaries in the vision data. This synchronization strategy ensures that both modalities are temporally aligned, providing a coherent basis for joint feature extraction and subsequent learning.

#### 3.2.2. Feature Projection

After preprocessing, the WiFi and vision data are prepared for joint representation learning. A fundamental challenge, however, arises from the inherent heterogeneity between the two modalities. WiFi data are typically structured as two-dimensional temporal sequences, whereas vision data contain three-dimensional spatiotemporal information. Without proper transformation, this mismatch hinders effective fusion.

To address this issue, modality-specific encoders are employed to transform both WiFi and vision signals into a unified latent feature space. After encoding, the WiFi features are flattened into 29,232-dimensional vectors for DUAL-Net and 37,296-dimensional vectors for MM-Fi, while the vision features are flattened into 9152-dimensional vectors. These flattened features are projected into 128-dimensional embeddings through modality-specific projection heads. The architectural details of these encoders are summarized in Table 1 and Table 2, which outline their respective layer configurations. Each encoder consists of convolutional blocks, optionally followed by pooling, and concludes with batch normalization and ReLU activation. This design ensures that both modalities are represented in a compatible format, providing a foundation for cross-modal alignment and joint learning.

Within this shared space, a feature enhancement strategy is introduced to improve representation expressiveness and strengthen generalization. As formalized in Equation (1), the encoding function Enc maps the input features *x* into *n* enhanced features. Here, *m* denotes the number of modalities, $\alpha_{jk}$ are randomly sampled weights between zero and one, and $z_{k}^{i}$ represents the enhanced features, where *i* indexes the *i*-th sample. This mechanism introduces controlled diversity into the enhanced features, enabling the network to capture richer semantic information beyond what raw encoders provide. By enriching the representation space in this way, it establishes a more discriminative and well-aligned foundation, which is essential for the success of the contrastive learning process in the next stage.

(1) $$z_{k}^{i}=Enc\left(x_{1}^{i},\cdots ,x_{m}^{i}\right)=\sum_{j=1}^{m}\alpha_{jk}x_{j}^{i},k=1,\cdots ,n.$$

#### 3.2.3. Contrastive Learning

To further refine the fused representations derived from WiFi and vision data, a contrastive learning strategy is employed to enhance feature discriminability across gesture categories. Building on the unified and semantically enriched feature space established during feature projection, contrastive learning strengthens decision boundaries by promoting semantically consistent clustering of features. While contrastive learning can be formulated by constructing positive pairs from different views of the same sample and negative pairs from different samples, such an instance-discrimination paradigm does not explicitly exploit semantic relationships among gesture categories. As a result, samples corresponding to the same gesture may remain dispersed in the representation space when variations in orientation, environment, or user-specific execution patterns are present. To learn gesture representations that are more robust to these variations, we employ supervised contrastive learning, where class labels are incorporated to guide feature alignment.

Specifically, fused features belonging to the same gesture category are encouraged to cluster together, whereas features from different categories are driven apart. This objective enhances intra-class consistency and inter-class discriminability in the shared latent space. The contrastive learning objective is defined as follows:

$$L_{CL}=\sum_{i\in I}\frac{-1}{\left|P(i)\right|}\sum_{p\in P(i)}log\frac{exp(z^{i}\cdot z^{p}/\tau )}{\sum_{a\in A(i)}exp\left(z^{i}\cdot z^{a}/\tau \right)},$$

here, $z^{i}$ and $z^{p}$ denote normalized features, · represents the inner product, τ is a temperature parameter, P(i) denotes the set of indices of positive samples that belong to the same class as the anchor *i*, and A(i) is the set of all other samples in the batch excluding *i*.

**Figure 3:**
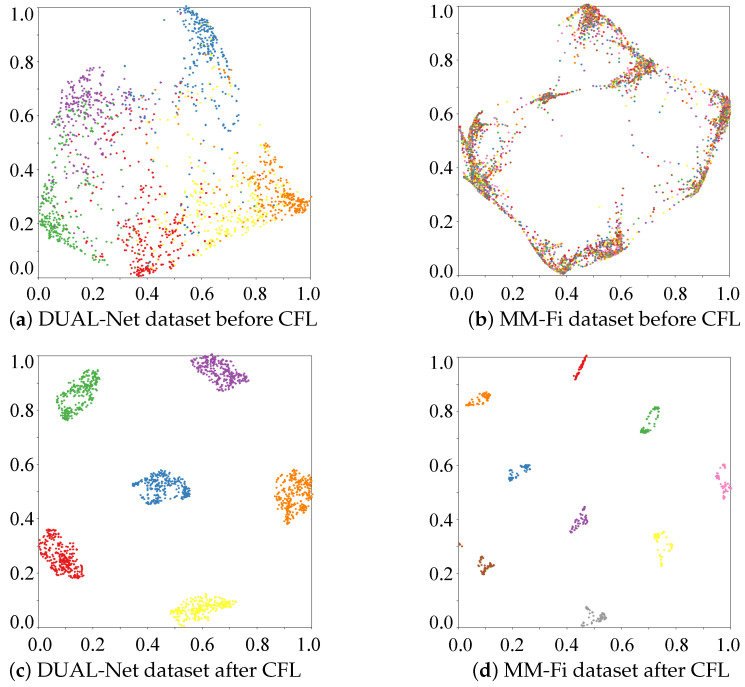
t-SNE visualization of fused gesture features before and after contrastive fusion learning (CFL) on the DUAL-Net and MM-Fi datasets. Different colors represent different gesture categories.

To evaluate the effectiveness of this process, we visualize the learned feature distributions using t-SNE. As shown in Figure 3, each point corresponds to a gesture feature, with different colors indicating different classes. After contrastive training, features belonging to the same class form compact clusters, while clusters from different classes are clearly separated. This improved organization provides strong evidence that contrastive learning enhances semantic alignment across modalities and increases the discriminative power of the fused feature space.

### 3.3. Cross-Modal Generation Module

**Figure 4:**
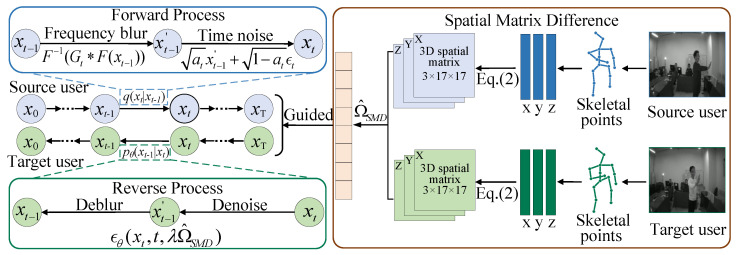
Illustration of the SMD-guided diffusion-based cross-modal generation module. The normalized SMD score is used as a structural condition to guide the reverse denoising process for user-adaptive WiFi generation.

As shown in Figure 4, the cross-modal generation (CMG) module aims to generate user-adaptive WiFi representations for unseen target users. The module is built upon a diffusion-based generative framework that progressively transforms source-user WiFi representations into target-user-adaptive features through a conditional denoising process. To ensure that the generated representations preserve user-specific characteristics, the proposed spatial matrix difference (SMD) is introduced as a structural conditioning signal. Specifically, skeletal information is encoded into spatial matrices to characterize structural variations between source and target users. The resulting SMD is then incorporated into the diffusion model to guide the generation process, enabling controllable synthesis of WiFi features that are consistent with the target user’s motion patterns.

#### 3.3.1. Forward Process

The forward process in the diffusion model progressively corrupts the original WiFi signal by injecting noise, ultimately transforming it into a pure noise distribution. WiFi data can be represented as a two-dimensional tensor, where the horizontal axis corresponds to the temporal sequence and the vertical axis corresponds to subcarriers in the frequency domain. This structure encodes both time-domain dynamics and frequency-domain characteristics. Unlike image data, WiFi signals contain richer structural dependencies across these two domains, which necessitates a tailored noise design strategy to model their signal distribution more precisely.

To preserve this structure during noise injection, we incorporate perturbations in both the frequency and time domains. First, we apply Gaussian convolution in the frequency domain to suppress high-frequency components and achieve progressive spectral smoothing:

$$G_{t}*F\left(x_{t-1}\right),$$

where $G_{t}$ is the Gaussian convolution kernel and ∗ denotes the convolution operation. Here, F(·) represents the Fourier transform, which maps the WiFi signal from the time domain to the frequency domain, enabling direct manipulation of its spectral components. The blurred frequency-domain signal is then transformed back to the time domain through the inverse Fourier transform:

$$x_{t-1}^{\prime }=F^{-1}\left(G_{t}*F\left(x_{t-1}\right)\right).$$

This operation ensures that high-frequency variations are effectively smoothed while preserving the overall temporal structure. Next, Gaussian noise is added in the time-domain to further degrade the signal and gradually push it toward a noise distribution:

$$x_{t}=\sqrt{\alpha_{t}}x_{t-1}^{\prime }+\sqrt{1-\alpha_{t}}\epsilon_{t},$$

where $\alpha_{t}$ is a predefined noise schedule and $\epsilon_{t}$ denotes Gaussian noise. As *t* increases, the signal converges toward Gaussian noise while maintaining structured degradation.

Finally, after repeated application of frequency-domain blurring and time-domain noise injection, the resulting $x_{t}$ follows a distribution formed by the mixture of the clean signal $x_{0}$ and Gaussian noise [50]:

$$q\left(x_{t}|x_{0}\right)∼N\left(x_{0};\mu_{t},\sigma_{t}^{2}I\right).$$

This frequency–time forward process provides a structured noise schedule that preserves signal properties at early steps and enables controllable degradation, laying a principled foundation for the reverse denoising process.

#### 3.3.2. Reverse Process

The reverse process, as the inverse of the forward corruption process, aims to progressively recover the clean WiFi data distribution from noisy observations through a parameterized denoising transformation. Whereas the forward process gradually corrupts the original signal with Gaussian perturbations in both the time and frequency domains, the reverse process learns the inverse mapping to reconstruct the underlying activity semantics and structural consistency. To formally realize this reconstruction, the learning objective is defined to align the model distribution with the true data distribution. Formally, the generative objective is to approximate the true data distribution $q(x_{0})$ with a learnable model $p_{\theta }\left(x_{0}\right)$ by minimizing their Kullback–Leibler (KL) divergence:

$$\theta^{*}=arg\underset{\theta }{min}D_{KL}\left(q\left(x_{0}\right)\|p_{\theta }\left(x_{0}\right)\right).$$

Direct optimization of this divergence is intractable since $q(x_{0})$ is unknown. To make the problem tractable, we adopt the diffusion probabilistic framework introduced by Ho et al. [51] and extended in DDIM [52]. This framework defines a forward diffusion process and a corresponding reverse denoising process, whose joint distributions can be factorized as:

$$\begin{array}{c} q\left(x_{0:T}\right)=q\left(x_{0}\right)\prod_{t=1}^{T}q\left(x_{t}|x_{t-1}\right),p_{\theta }\left(x_{0:T}\right)=p\left(x_{T}\right)\prod_{t=1}^{T}p_{\theta }\left(x_{t-1}|x_{t}\right), \end{array}$$

The forward process forms a linear Gaussian Markov chain that progressively injects noise into the data, while the reverse process aims to invert this transformation through a learnable denoising distribution.

To address the intractability of optimizing the global KL divergence, we apply the variational inference formulation [51], which decomposes the overall objective into a sum of stepwise KL divergences between the true posterior and the parameterized reverse transitions:

$$\begin{array}{c} D_{KL}\left(q\left(x_{0}\right)\|p_{\theta }\left(x_{0}\right)\right)=E_{q(x_{0:T})}\left[\sum_{t=1}^{T}D_{KL}\left(q\left(x_{t-1}|x_{t},x_{0}\right)\|p_{\theta }\left(x_{t-1}|x_{t}\right)\right)\right]+C. \end{array}$$

This decomposition transforms a single intractable global objective into a set of manageable local objectives at each timestep, simplifying training and improving stability. Accurately modeling the conditional posterior $q(x_{t-1}|x_{t},x_{0})$ at each step is key to ensuring that the learned reverse process $p_{\theta }\left(x_{0:T}\right)$ converges to the true data distribution.

**Gaussian Posterior Approximation.** Following the DDPM formulation, we approximate the reverse process using Gaussian transitions. Under this approximation, the posterior distribution at each diffusion step can be expressed as:

$$q\left(x_{t-1}|x_{t},x_{0}\right)=N\left(x_{t-1};{\tilde{\mu }}_{t-1},{\tilde{\sigma }}_{t-1}^{2}I\right),$$

where ${\tilde{\mu }}_{t-1}=\frac{1}{{\bar{\gamma }}_{t}^{2}}\left(\gamma_{t}{\bar{\gamma }}_{t-1}^{2}x_{t}+\gamma_{t-1}\sigma_{t}^{2}x_{0}\right),{\tilde{\sigma }}_{t-1}=\frac{{\bar{\gamma }}_{t-1}}{{\bar{\gamma }}_{t}}\sigma_{t}.$

The mean term ${\tilde{\mu }}_{t-1}$ represents an adaptive combination of the noisy sample $x_{t}$ and the clean signal $x_{0}$, weighted according to the noise variance $\sigma_{t}^{2}$. This formulation yields a closed-form posterior that provides an optimal intermediate estimate of $x_{t-1}$ conditioned on both observed and clean signals.

**Learned Reverse Distribution.** Building on the posterior formulation introduced earlier, the learned reverse process aims to approximate the true posterior during inference. To this end, the model defines a parameterized Gaussian transition:

$$p_{\theta }\left(x_{t-1}|x_{t}\right)=N\left(x_{t-1};\mu_{\theta }\left(x_{t}\right),\sigma_{\theta }^{2}\left(x_{t}\right)I\right),$$

where both the mean $\mu_{\theta }\left(x_{t}\right)$ and the variance $\sigma_{\theta }\left(x_{t}\right)$ are predicted by a neural network. The training objective minimizes the KL divergence between the true posterior and the learned reverse distribution [50]:

$$\begin{array}{c} D_{KL}\left(q\left(x_{t-1}|x_{t},x_{0}\right)\|p_{\theta }\left(x_{t-1}|x_{t}\right)\right)=E_{q(x_{0})}\left[\frac{1}{2{\tilde{\sigma }}_{t-1}^{2}}{∥{\tilde{\mu }}_{t-1}-\mu_{\theta }\left(x_{t}\right)∥}^{2}\right]. \end{array}$$

When the model variance is fixed to match the posterior variance $\sigma_{\theta }\left(x_{t}\right)={\tilde{\sigma }}_{t-1}$, this divergence simplifies to a weighted mean squared error (MSE) between the ground-truth and predicted means. This formulation not only reduces computational complexity but also preserves theoretical consistency with the DDPM framework [50,51]. Moreover, this learned reverse distribution serves as the foundation for reconstructing clean RF signals in the generative denoising process, thereby ensuring accurate and stable signal restoration.

#### 3.3.3. Conditional Guidance with SMD

Generating user-adaptive WiFi representations from RF signals alone is challenging, since WiFi measurements encode human motion implicitly through signal reflections. User-specific factors, including body proportions, limb lengths, gesture amplitudes, and execution patterns, are weakly encoded in the RF domain. However, they substantially affect propagation paths and multipath reflections. Therefore, the same gesture performed by different users can induce distinct CSI patterns. Modeling such variations requires additional structural guidance beyond RF signals.

To address this issue, we introduce the spatial matrix difference (SMD) as a cross-modal structural prior. The key idea is that variations in skeletal structure and motion trajectories are reflected in both skeletal representations and the corresponding WiFi signal patterns. By measuring structural differences across users, SMD provides a surrogate measure of user-specific variations in WiFi responses. This prior is incorporated into the diffusion process to guide the generation of user-adaptive WiFi representations.

During target-user adaptation, the proposed DUAL-Net framework does not require any WiFi samples from the target user. Instead, it utilizes a short RGB video of the target user performing a representative gesture, which is easily obtainable in practice and does not require dedicated WiFi data collection. The video provides visual observations for capturing user-specific motion characteristics while avoiding additional target-user WiFi sampling.

To obtain reliable skeletal representations, we employ VideoPose3D [53] to estimate 3D skeletal keypoints. Compared with conventional pose-estimation approaches, VideoPose3D preserves temporal consistency while maintaining computational efficiency. Each frame contains 17 keypoints, which are organized into skeletal matrices that encode pairwise structural relationships among body joints. For each spatial dimension (x,y,z), we compute a 17×17 matrix in which each element captures the relative position between keypoints. As shown in Equation (2), i,j∈[1,2,…,17] denote keypoint indices, and *X* represents the spatial matrix for the *x*-dimension (size 1×17×17), with *Y* and *Z* defined analogously.

(2) $$X=\left\{\begin{array}{c} x_{i}-x_{j},\\ x_{i}, \end{array}\right.\begin{array}{c} i\neq j;\\ i=j. \end{array}\;Y=\left\{\begin{array}{c} y_{i}-y_{j},\\ y_{i}, \end{array}\right.\begin{array}{c} i\neq j;\\ i=j. \end{array}\;Z=\left\{\begin{array}{c} z_{i}-z_{j},\\ z_{i}, \end{array}\right.\begin{array}{c} i\neq j;\\ i=j. \end{array}$$

To guide the cross-user generation process, it is crucial to quantitatively measure the discrepancy between the skeletal matrices of source and target users. We adopt the mean squared error (MSE) as the metric, since it provides a sensitive and differentiable measure of structural differences. Specifically, MSE captures the element-wise squared deviations between the spatial matrices of source and target users, effectively quantifying user-specific variations in body structure and gesture execution. The SMD is defined as:

$$\Omega_{SMD}=\frac{1}{n}\left(|\right|X_{t}-X_{s}{\left|\right|}^{2}+\left|\right|Y_{t}-Y_{s}{\left|\right|}^{2}+\left|\right|Z_{t}-Z_{s}\left|{|}^{2}\right),$$

where *X*, *Y*, *Z* represent the skeletal matrices in the three spatial dimensions, *s* and *t* refer to the source and target users, and n=3 denotes the number of spatial dimensions. This formulation encourages the generated WiFi data to retain target user-specific spatial semantics derived from vision guidance.

Since the magnitude of $\Omega_{SMD}$ may vary across user pairs, the score is normalized to ensure numerical stability:

$${\hat{\Omega }}_{SMD}=\frac{\Omega_{SMD}-\Omega_{\min}}{\Omega_{\max}-\Omega_{\min}+\epsilon },$$

where ϵ is a small constant introduced to avoid division by zero. The normalized SMD score lies within the range [0,1] and serves as the structural conditioning variable for the diffusion model.

To incorporate user-specific structural information into the generation process, the normalized SMD score is introduced as a structural condition for the diffusion model. The rationale is that skeletal discrepancies capture variations in body geometry and motion execution, which are closely associated with user-dependent WiFi responses. By conditioning the denoising process on this structural prior, the model is encouraged to generate WiFi representations that better reflect the characteristics of the target user.

Specifically, the normalized SMD score is first projected into a latent embedding and then incorporated into the denoising network as a conditioning signal. This projection maps the scalar SMD score into the same latent conditioning space as the diffusion timestep embedding, ensuring dimensional compatibility during denoising. The noise prediction network is therefore reformulated as:

$$\epsilon_{\theta }\left(x_{t},t,\lambda {\hat{\Omega }}_{SMD}\right),$$

where $x_{t}$ denotes the noisy WiFi representation at diffusion step *t*, ${\hat{\Omega }}_{SMD}$ denotes the normalized structural discrepancy derived from the source and target skeletal representations, and λ controls the contribution of the structural condition.

Accordingly, the conditional reverse distribution is defined as:

$$p_{\theta }\left(x_{t-1}|x_{t},{\hat{\Omega }}_{SMD}\right)=N\left(x_{t-1};\mu_{\theta }\left(x_{t},{\hat{\Omega }}_{SMD}\right),\sigma_{t}^{2}I\right)$$

where the mean of the reverse transition is conditioned on the structural prior. The corresponding training objective is:

$$L_{CMG}=E\left[{∥\epsilon -\epsilon_{\theta }\left(x_{t},t,\lambda {\hat{\Omega }}_{SMD}\right)∥}_{2}^{2}\right]$$

Through this formulation, the SMD condition explicitly injects user-specific structural cues into the generation process, enabling the synthesized WiFi representations to better adapt to target-user characteristics. The coefficient λ is a standard guidance strength hyperparameter that controls the contribution of the SMD-based structural condition in the diffusion process. It is not a learned parameter but is selected based on a held-out validation set. In practice, we perform a lightweight grid search over a small range and observe that the model exhibits stable performance within a relatively wide interval, indicating that the proposed method is not sensitive to the exact choice of λ. This behavior is consistent with prior diffusion-based conditional generation methods, where similar guidance-scale parameters are typically tuned in the same manner.

### 3.4. Gesture Recognition Module

The gesture recognition module integrates domain-invariant and user-adaptive features for final classification. The domain-invariant representation $h_{env}$ is obtained from the contrastive fusion learning module, which provides stable gesture semantics across different environments. The user-adaptive representation $h_{usr}$ is derived from the WiFi features synthesized by the cross-modal generation module, which introduces user-specific structural and motion characteristics. These two representations provide complementary cues for recognition: $h_{env}$ improves robustness against environmental changes, while $h_{usr}$ compensates for inter-user variations.

The fused representation is computed as $c=g(\left[h_{env},h_{usr}\right]),$ and the final prediction is $\hat{y}=Softmax\left(Wc\right),$ where *W* denotes the classification layer. In this formulation, the fusion function g(·) serves to combine the complementary information from the two branches before classification, allowing the classifier to exploit both environment-robust and user-adaptive cues.

We adopt a training strategy under the two-stage learning framework of DUAL-Net, separating offline representation learning from online inference.

**Encoder Update.** In this stage, both real and CMG-generated WiFi samples are used to update the encoders, while the classifier is kept fixed. This encourages the encoders to incorporate user-adaptive information from CMG-generated features while preserving domain-invariant representations learned from contrastive fusion learning.

**Classifier Update.** In this stage, the encoders are frozen and the classifier is optimized using both real and generated samples under a cross-entropy loss:

$$L_{CE}=-\sum_{i}^{L}y_{i}\log{\hat{y}}_{i}.$$

This step refines the decision boundary by jointly considering stable gesture semantics and user-specific variations introduced by the generated samples. Overall, this alternating optimization strategy implements the two-stage learning paradigm of DUAL-Net, where CFL and CMG are performed during offline learning, while online inference remains lightweight with reduced computational overhead.

## 4. Experimental Setup

### 4.1. Datasets

While gesture recognition has achieved significant advances in both WiFi and vision sensing, publicly available datasets that provide synchronized multimodal data remain limited. This limitation presents a challenge for developing and evaluating robust models that require the integration of heterogeneous modalities. To facilitate a comprehensive evaluation of the proposed DUAL-Net framework, we construct a new multimodal dataset, referred to as the DUAL-Net dataset, which comprises synchronized WiFi Channel State Information (CSI) and RGB video data. The dataset is collected under varied environmental conditions and across a diverse set of users, with all participants providing informed consent. This makes it particularly suitable for studying cross-modal fusion, user-adaptive modeling, and domain-invariant representation learning.

In addition to our self-collected dataset, we employ the publicly available MM-Fi dataset [54], which includes five sensing modalities—WiFi CSI, RGB video, depth video, LiDAR, and mmWave radar—and covers a wide range of user behaviors in multiple environments. For this study, we focus on the WiFi CSI and RGB video components to ensure consistency in modality alignment. Evaluation on both the DUAL-Net and MM-Fi datasets demonstrates consistent performance across users and environments. A detailed summary of the two datasets used in our experiments is provided in Table 3.

**Figure 5:**
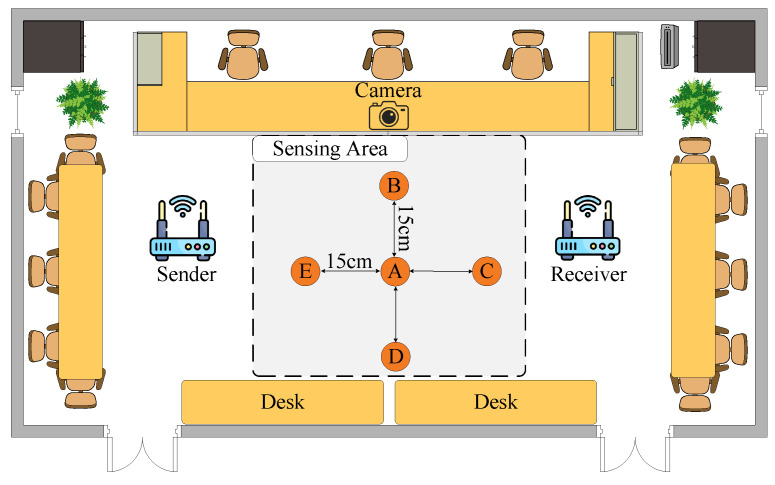
Multimodal data collection setup for the DUAL-Net dataset.

**Figure 6:**
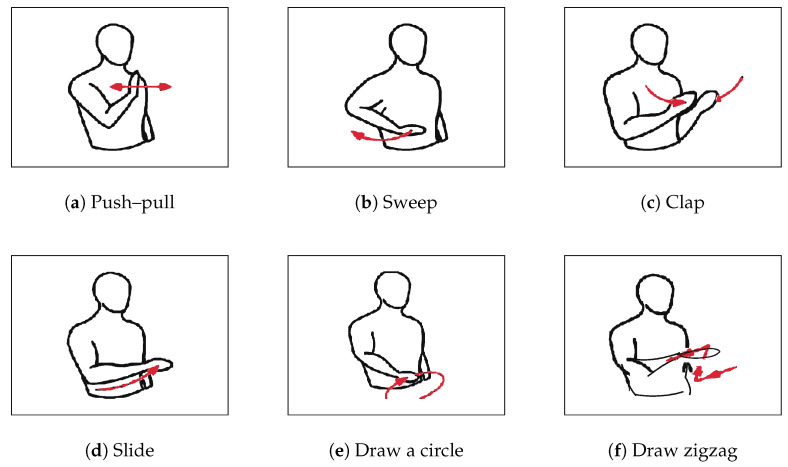
Illustrations of the six gesture categories in the DUAL-Net dataset, the arrows indicate the gesture directions.

**DUAL-Net.** As illustrated in Figure 5, the DUAL-Net dataset was designed to evaluate the proposed framework across different environments and users. It contains six gesture categories, as shown in Figure 6, namely *Push–pull, Sweep*, *Clap*, *Slide*, *Draw a circle*, and *Draw zigzag*. The data are collected from 10 volunteers (including 3 females) at five locations labeled A, B, C, D, and E. These gestures cover both simple directional movements and complex trajectory-based motions, while the five locations represent different spatial configurations that may affect gesture recognition. RGB video data was captured using a Sony FDR-AX60 camera (Sony Corporation, Tokyo, Japan) from different perspectives, and WiFi CSI was collected using two PCs equipped with Intel 5300 NICs (Intel Corporation, Santa Clara, CA, USA) as the transmitter and receiver. Each device had three antennas, and each antenna pair recorded 30 subcarriers, resulting in 270 subcarriers for each WiFi sample (3antennas×3antennapairs×30subcarriers). For each location, six samples were collected per gesture category and per volunteer, resulting in 1800 samples in total (6gesturecategories×5locations×6samples×10volunteers).

**MM-Fi.** The MM-Fi dataset provides a broad testbed for gesture recognition under varied environments and user profiles. It comprises five sensing modalities collected in four environments (E01–E04), with 10 participants in each environment performing 27 activities, nine of which are gesture-specific. We consider nine gesture categories covering arm stretching, arm extension, arm raising, and waving motions, including *horizontal arm stretch*, *vertical arm stretch*, *left arm extension*, *right arm extension*, *simultaneous arm extension*, *left arm raise*, *right arm raise*, *left-hand wave*, and *right-hand wave*. Each activity was recorded for 297 frames, and only RGB video and WiFi CSI are used in our experiments. RGB video was captured using a high-precision camera with a resolution of 640×480 pixels, providing detailed visual information for gesture recognition. WiFi CSI was captured using a TP-Link N750 router (TP-Link Technologies Co., Ltd., Shenzhen, China) and the Atheros CSI tool (Atheros 802.11n PCI/PCI-E chips) on the 5 GHz band with 40 MHz bandwidth. Each WiFi sample consists of 297 frames from 3 antenna pairs, with each pair recording 114 subcarriers, resulting in a CSI representation of 297×3×114 per sample. In total, the selected MM-Fi gesture subset contains 8640 samples (9gestures×4environments×24samples×10volunteers).

### 4.2. Implementation

The proposed DUAL-Net framework was implemented in Python 3.8 using PyTorch v1.8 as the deep learning backend. PyTorch was selected for its flexibility and efficient support for multimodal data processing. All experiments were conducted on a workstation equipped with an Intel Xeon E5-2698 v4 CPU (Intel, Santa Clara, CA, USA), four NVIDIA Tesla V100 GPUs (Nvidia, Santa Clara, CA, USA), and 256 GB RAM, running the Ubuntu 20.04 operating system. For the cross-modal generation module, the diffusion-based generator was optimized using AdamW with a learning rate of 1 $\times \;10^{-3}$ and a batch size of 16. The number of diffusion steps was set to 100. The noise schedule ranged from 1 $\times \;10^{-4}$ to 0.003, and the blur variance was set to 1 $\times \;10^{-10}$. After training, the generated target-adaptive WiFi samples were saved offline and then used in the subsequent gesture recognition stage. For contrastive fusion learning, the model was trained using SGD with a learning rate of 0.01, momentum of 0.9, weight decay of 1 $\times \;10^{-4}$, and a batch size of 32. The training process lasted for 400 epochs, and the temperature parameter in the contrastive loss was set to 0.07. For gesture recognition, the recognition network was optimized using cross-entropy loss. The learning rate of the encoder part was set to 1 $\times \;10^{-4}$, while the learning rate of the classifier part was set to 1 $\times \;10^{-3}$. The batch size was set to 64, and the model was trained for 400 epochs. Each experiment was repeated ten times, and the average recognition accuracy was reported.

### 4.3. Experimental Notation

In the experiments, DUAL-Net denotes the full proposed framework. CFL denotes contrastive fusion learning, CMG denotes cross-modal generation, and SMD denotes spatial matrix difference. The abbreviations “w/” and “w/o” indicate “with” and “without”, respectively. In the ablation studies, w/o CFL removes the contrastive fusion learning module, w/o CMG removes the cross-modal generation module, and w/o SMD removes the SMD guidance from the generation process.

## 5. Experimental Results

This section presents a comprehensive evaluation of the proposed DUAL-Net framework. The experiments include overall performance assessment in cross-user and cross-environment scenarios, comparative analysis against multimodal and single-modality methods, ablation studies examining the roles of the contrastive fusion learning and cross-modal generation modules, evaluation of the recognition performance of generated WiFi data, and analysis of training dynamics, including the impact of synthesized sample quantity on cross-user accuracy and the convergence behavior of training loss. The results demonstrate that DUAL-Net achieves robust and scalable gesture recognition across diverse users and environments.

### 5.1. Overall Performance Evaluation of DUAL-Net

**(1) Cross-user evaluation:** In this experiment, we assess DUAL-Net’s performance in cross-user scenarios, specifically evaluating its ability to generalize across different users. To rigorously examine the model’s generalization capabilities, we conduct evaluations on both the DUAL-Net dataset and the MM-Fi dataset. For each dataset, the model is trained using gesture data from all but one user, who serves as the target for testing. This process is repeated for each user, ensuring comprehensive evaluation across all possible cross-user scenarios.

**Figure 7:**
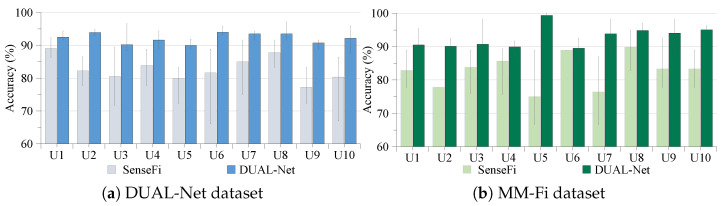
Cross-user evaluation results on the DUAL-Net and MM-Fi datasets, highlighting DUAL-Net’s ability to generalize across unseen users.

As shown in Figure 7, we evaluate the robustness and consistency of DUAL-Net using min-max bars, which indicate the maximum and minimum recognition accuracies obtained from ten randomly repeated experiments. To establish a fair comparison, we also compare with SenseFi [29], a widely adopted deep learning benchmark framework for WiFi human sensing. The results demonstrate that DUAL-Net achieves consistently high recognition accuracy with unseen users, reflecting its strong generalization ability in cross-user scenarios. While recognition accuracy typically exceeds 90%, a few cases fall below this threshold due to the inherent complexity of modeling diverse user characteristics. Nevertheless, DUAL-Net exhibits strong adaptability, achieving up to 100% accuracy in certain instances. Compared with SenseFi, DUAL-Net achieves consistent accuracy improvements, with average improvements of 9.52% and maximum improvements of 24.38% across different experimental settings, thereby confirming its effectiveness in enhancing cross-user gesture recognition.

**(2) Cross-environment evaluation:** In this experiment, we assess DUAL-Net’s performance across varying environmental conditions, focusing on its ability to adapt to different locations and environmental setups. The evaluation is conducted on two datasets, the DUAL-Net dataset, which includes data collected from multiple locations, and the publicly available MM-Fi dataset, which includes data from various environmental deployments. For each dataset, we use one environment (or location) as the test set, while the remaining environments (or locations) are used for training. This approach ensures that DUAL-Net is evaluated for its ability to generalize across different settings, a critical factor for real-world deployment.

**Figure 8:**
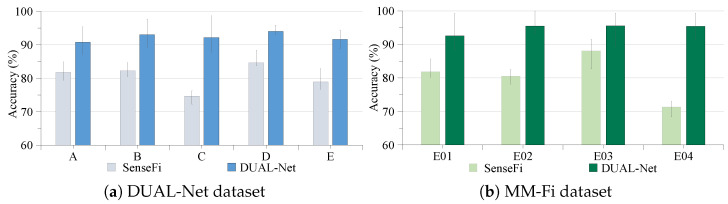
Gesture recognition accuracy across different locations on the DUAL-Net dataset and varying environmental conditions on the MM-Fi dataset.

As shown in Figure 8, the experimental results demonstrate that DUAL-Net consistently achieves over 90% gesture recognition accuracy on both the DUAL-Net and MM-Fi datasets. The relatively lower performance observed in the cross-location task on the DUAL-Net dataset is primarily due to the limited number of samples available at each location, which constrains the model’s ability to generalize across spatial variations. Nevertheless, DUAL-Net maintains strong robustness across diverse environments, confirming its adaptability to spatial and environmental changes. Furthermore, when compared with SenseFi [29], a widely adopted deep learning benchmark framework for WiFi human sensing, DUAL-Net achieves average accuracy improvements of 11.93% on the DUAL-Net dataset and 14.35% on the MM-Fi dataset, thereby demonstrating its superior capability for robust cross-environment gesture recognition.

### 5.2. Comparative Analysis of DUAL-Net

**(1) Comparison with multimodal learning methods:** To assess the effectiveness of the proposed DUAL-Net framework, we conducted an extensive comparative analysis against four state-of-the-art multimodal fusion methods: Attnsense [55], Deepsense [56], Concat [57], and Cosmo [58]. To ensure a fair comparison, all multimodal baselines are evaluated under the same dataset, train/test splits, and evaluation metrics. We strictly follow their original implementations and reproduce them under consistent software and hardware environments. For multimodal consistency, all methods use both WiFi and vision inputs when applicable, and identical preprocessing and augmentation strategies are applied where possible.

**Figure 9:**
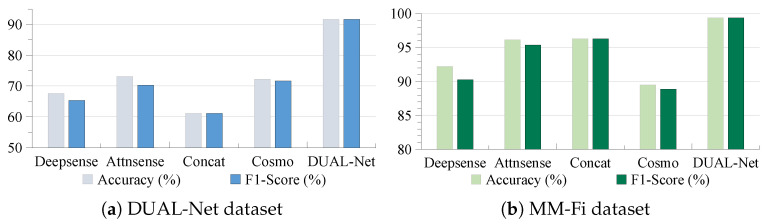
Comparison of DUAL-Net and multimodal baselines on the DUAL-Net and MM-Fi datasets.

As shown in Figure 9, DUAL-Net consistently outperforms these methods on both the DUAL-Net and MM-Fi datasets. Specifically, DUAL-Net achieved recognition accuracy exceeding 90% across both datasets, with relative improvements of 18.52% and 3.08% over the strongest baseline, respectively. Moreover, DUAL-Net yields substantial F1-score gains (up to 30.57%), indicating its superior ability to differentiate between gesture categories and mitigate misclassification errors. These quantitative results highlight DUAL-Net’s robust generalization capability across diverse users and environments, establishing it as a reliable and competitive framework for real-world gesture recognition applications.

**(2) Comparison with single-modality methods:** To comprehensively evaluate the effectiveness of DUAL-Net in heterogeneous multimodal fusion, we conducted comparative experiments on both the DUAL-Net and MM-Fi datasets against two representative single-modality gesture recognition methods, namely WiGr [59], a WiFi-based approach, and Snapture [60], a vision-based approach. WiGr employs a two-path prototype network for WiFi-based cross-domain gesture recognition, while Snapture integrates both static and dynamic gestures for vision-based recognition. These baselines represent state-of-the-art methods in their respective modalities, providing strong benchmarks for evaluation. For single-modality baselines, we maintain the same evaluation protocol as multimodal methods, while restricting inputs to their corresponding modality (WiFi or vision) and ensuring that no cross-modal information is introduced.

**Figure 10:**
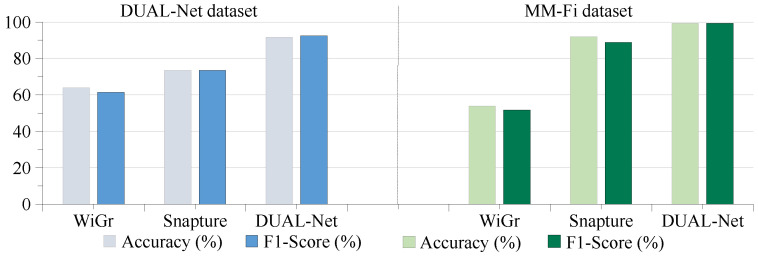
Comparison of DUAL-Net and single-modality baselines on the DUAL-Net and MM-Fi datasets.

As shown in Figure 10, DUAL-Net demonstrates clear superiority over both methods in terms of accuracy and F1-score across the two datasets. On the DUAL-Net dataset, DUAL-Net achieves relative gains of 27.74% in accuracy and 31% in F1-score compared to WiGr, and 18.21% in accuracy and 18.91% in F1-score compared to Snapture. On the MM-Fi dataset, similar improvements are observed, with accuracy and F1-score gains exceeding 45.51% and 47.63%, respectively. These results highlight the benefits of combining WiFi and vision signals, where DUAL-Net effectively leverages the complementary strengths of both modalities to enhance recognition performance. By jointly integrating WiFi’s robustness to environmental occlusion with vision’s fine-grained spatial representation, DUAL-Net overcomes the inherent limitations of single-modality approaches and achieves reliable performance across diverse sensing conditions.

### 5.3. Ablation Analysis of DUAL-Net

**Figure 11:**
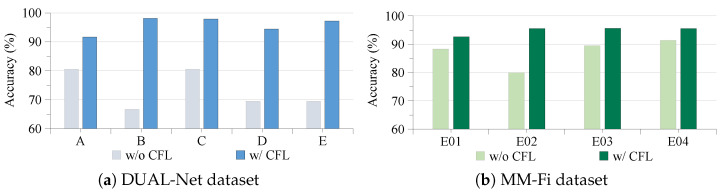
Recognition performance with and without CFL on the DUAL-Net and MM-Fi datasets.

**(1) Effectiveness of contrastive fusion learning:** To evaluate the impact of the Contrastive fusion learning (CFL) module, we performed ablation experiments on both the DUAL-Net and MM-Fi datasets. As shown in Figure 11, the configuration without the contrastive fusion learning module (w/o CFL) exhibits lower gesture recognition accuracy, while the inclusion of the module (w/ CFL) substantially enhances performance. Specifically, integrating contrastive fusion learning improves the average accuracy by 22.53% on the DUAL-Net dataset and 7.52% on the MM-Fi dataset.

These results demonstrate that the contrastive fusion learning module effectively strengthens multimodal fusion by promoting consistent feature representations across WiFi and vision modalities. By maximizing intra-class similarity and enforcing inter-class separability, the module strengthens the discriminability of multimodal features, thereby enabling the model to more effectively distinguish fine-grained gesture categories. Consequently, DUAL-Net achieves a more coherent and unified feature space, which translates into higher accuracy and greater robustness in cross-user recognition scenarios.

**Figure 12:**
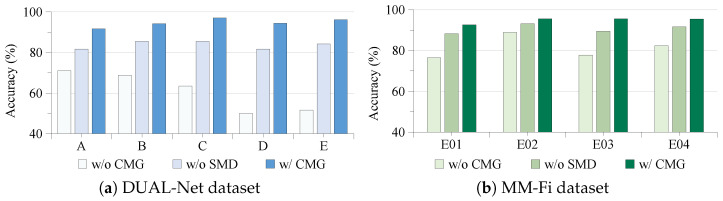
Recognition performance of the CMG module with and without SMD-guided conditioning on the DUAL-Net and MM-Fi datasets.

**(2) Effectiveness of cross-modal generation:** To evaluate the contribution of the cross-modal generation (CMG) module, we conducted ablation studies using different configurations of DUAL-Net. As shown in Figure 12, the model without CMG (w/o CMG) achieves the lowest recognition accuracy, indicating that the absence of cross-modal generation significantly limits the model’s ability to adapt to unseen users. In contrast, incorporating the full CMG module (w/ CMG), which generates WiFi representations guided by vision-based semantic priors, yields the highest performance. Specifically, the inclusion of CMG achieves up to 44.55% improvement on the DUAL-Net dataset and 17.82% improvement on the MM-Fi dataset, underscoring its effectiveness in producing user-adaptive features.

To further validate the role of cross-modal guidance, we compare the full CMG configuration with an alternative setting in which WiFi data are synthesized without structural conditioning (w/o SMD). The results show that removing SMD guidance leads to a performance degradation, demonstrating the importance of visual structural priors for user-adaptive WiFi generation.

### 5.4. Evaluation of Generated Data Performance

To evaluate the effectiveness of CMG, particularly the contribution of the proposed SMD-guided conditioning mechanism, we analyze generated WiFi representations from three complementary perspectives, including recognition performance, feature-level consistency, and signal-level consistency. First, we employ SenseFi [29] as an external gesture recognition model to compare WiFi samples generated without SMD guidance (w/o SMD), samples generated by the full CMG module (w/ CMG), and real WiFi samples used as a performance reference. Second, we visualize real, generated, and mixed WiFi features using t-SNE to examine feature distributions. Finally, we compare amplitude variations of real and generated WiFi signals to assess whether generated samples preserve gesture-related temporal dynamics. All evaluations are conducted on both the DUAL-Net and MM-Fi datasets.

**Figure 13:**
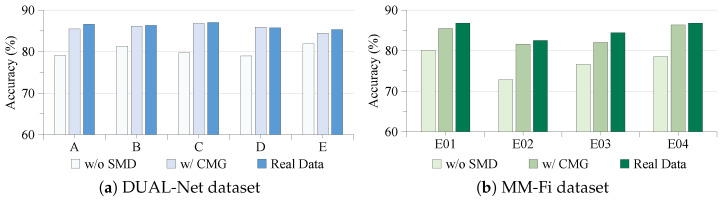
Accuracy comparison using WiFi data generated without SMD guidance (w/o SMD), generated by the complete CMG module (w/ CMG), and real data.

**(1) Recognition performance analysis:** As shown in Figure 13, WiFi samples generated with SMD guidance consistently outperform those generated without SMD guidance on both datasets. Specifically, recognition accuracy increases by up to 7.08% on the DUAL-Net dataset and 8.79% on the MM-Fi dataset. The results indicate that SMD guidance helps the diffusion model generate more useful WiFi features for unseen users. Although the accuracy is still lower than that obtained with real WiFi data, the generated samples preserve important gesture information and provide a practical alternative when target-user WiFi data are unavailable.

**Figure 14:**
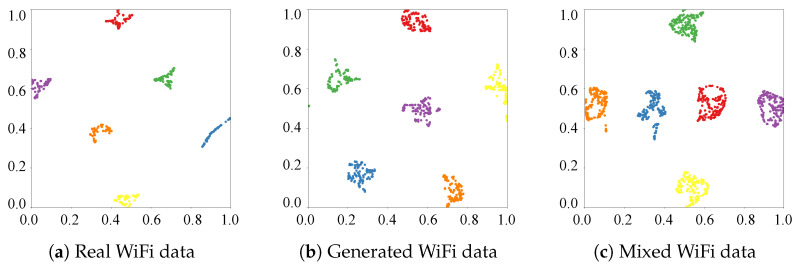
t-SNE visualization of WiFi feature embeddings for real, generated, and mixed data on the DUAL-Net dataset. Different colors represent different gesture categories.

**Figure 15:**
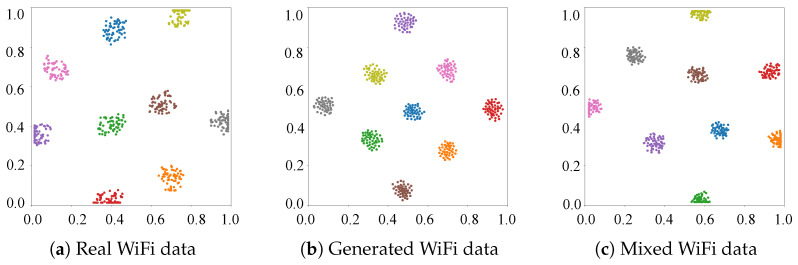
t-SNE visualization of WiFi feature embeddings for real, generated, and mixed data on the MM-Fi dataset. Different colors denote different gesture categories.

**(2) Feature-level consistency analysis:** To evaluate whether the generated WiFi data preserve gesture-related feature structures, we visualize the feature distributions of real, generated, and mixed WiFi samples using t-SNE. Figure 14 presents results on the DUAL-Net dataset, while Figure 15 reports results on the MM-Fi dataset. As shown in Figure 14a and Figure 15a, real WiFi samples form clear class-wise clusters on both datasets, indicating that CSI features contain discriminative gesture information. Figure 14b and Figure 15b further show that generated samples also exhibit separable class structures, suggesting that the CMG module preserves gesture-level semantic information during generation. When real and generated samples are jointly visualized in Figure 14c and Figure 15c, the mixed distributions remain compact within classes while maintaining clear inter-class separability. This indicates that generated features are well aligned with real WiFi representations and can serve as complementary training samples.

**Figure 16:**
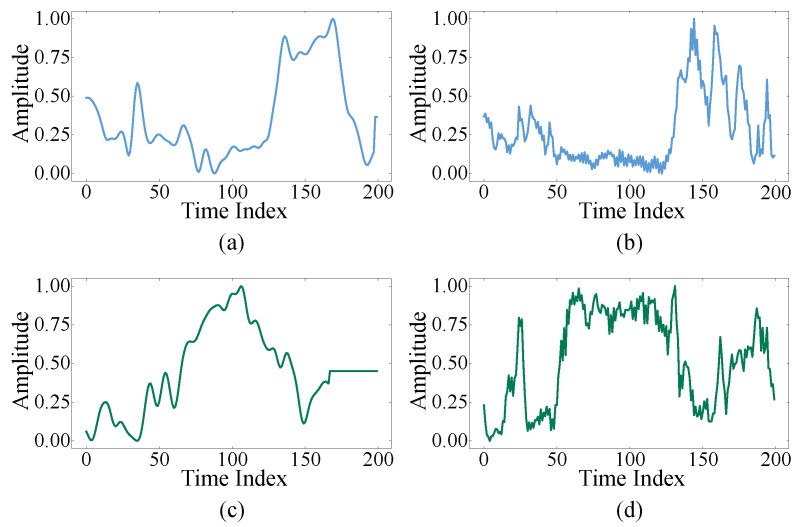
Amplitude variations of real and generated WiFi data on the DUAL-Net dataset. (**a**) Real WiFi data of user U1 for gesture G4. (**b**) Generated WiFi data of user U1 for gesture G4. (**c**) Real WiFi data of user U1 for gesture G1. (**d**) Generated WiFi data of user U1 for gesture G1.

**Figure 17:**
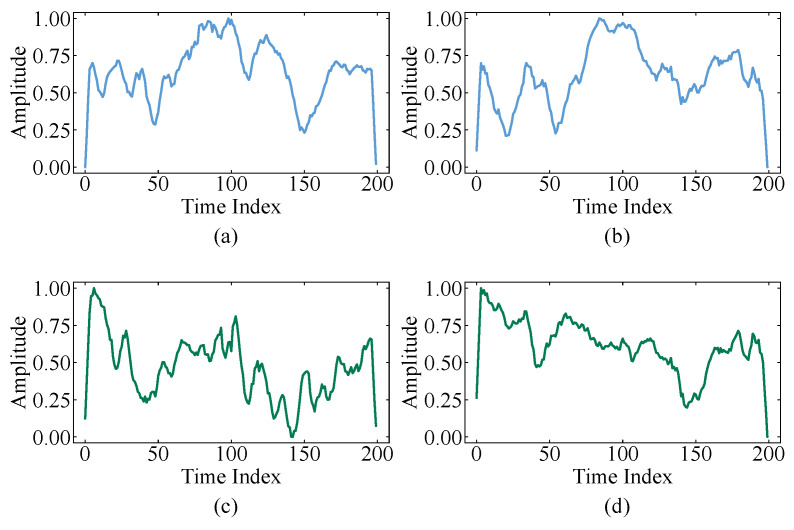
Amplitude variations of real and generated WiFi data on the MM-Fi dataset. (**a**) Real WiFi data of user U10 for gesture G2. (**b**) Generated WiFi data of user U10 for gesture G2. (**c**) Real WiFi data of user U10 for gesture G6. (**d**) Generated WiFi data of user U10 for gesture G6.

**(3) Signal-level consistency analysis:** We further analyze whether the generated WiFi signals preserve gesture-related temporal dynamics by comparing the amplitude variations of real and generated samples. Representative real–generated pairs from the DUAL-Net and MM-Fi datasets are shown in Figure 16 and Figure 17, respectively. As illustrated in Figure 16, the generated signals closely follow the dominant fluctuation patterns of the corresponding real signals, particularly in high-response regions. Similar trends can be observed in Figure 17, where the generated signals also preserve the main temporal variation patterns of real WiFi measurements. Although exact point-wise matching is not expected due to the stochastic nature of the generation process, the generated signals consistently retain key amplitude variations and local response structures associated with gesture-induced CSI dynamics. These results indicate that the CMG module generates WiFi signals that are consistent with real data at the level of dominant temporal patterns across both datasets.

### 5.5. Additional Experimental Analysis

To further examine the behavior of DUAL-Net beyond the main recognition results, we analyze its adaptation performance, training process, confusion matrix analysis, and computational cost. These analyses are intended to clarify how the number of generated samples affects cross-user recognition, whether the training process converges stably, which gesture categories remain difficult to distinguish, and whether the model can support efficient inference.

**Figure 18:**
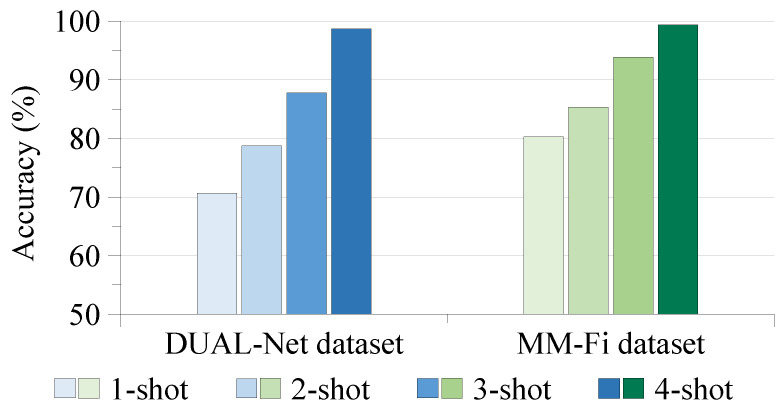
Cross-user accuracy with different numbers of generated WiFi samples.

**(1) Adaptation and training behavior.** Figure 18 shows the cross-user recognition accuracy obtained with different numbers of generated WiFi samples on the DUAL-Net and MM-Fi datasets. The accuracy generally increases as more generated samples are used, indicating that the generated data provide useful user-adaptive information when target-user WiFi samples are limited. The improvement becomes less pronounced as the number of generated samples increases, suggesting that a small number of generated samples already contributes a substantial part of the adaptation benefit.

**Figure 19:**
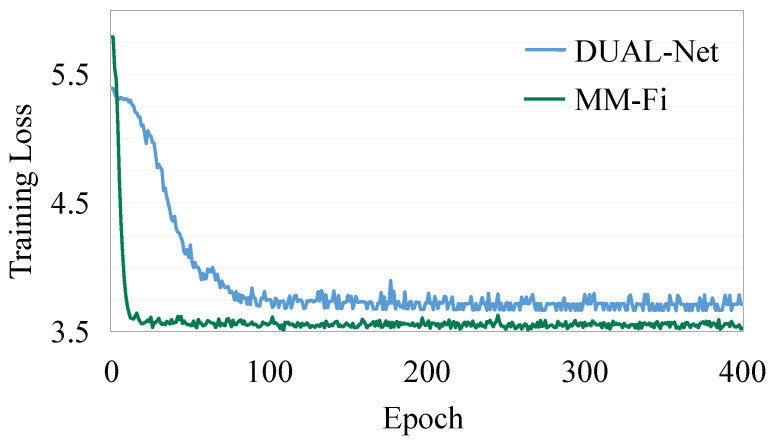
Training loss curves across different training epochs.

Figure 19 presents the training loss curves on the two datasets. The loss decreases steadily during training and gradually approaches a stable range, indicating that the optimization process remains stable under the adopted training strategy. This result also suggests that jointly using real and generated samples does not introduce obvious instability during training.

**Figure 20:**
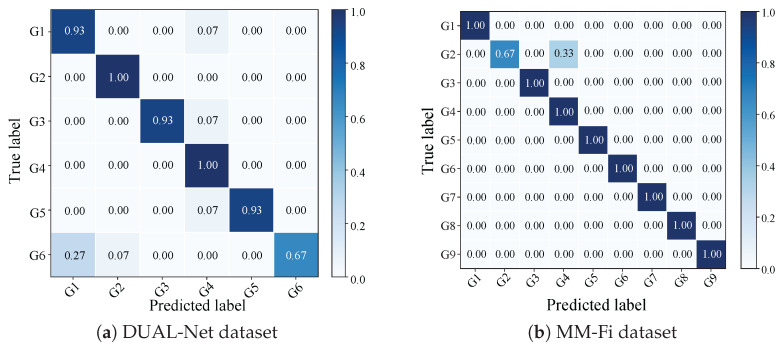
Confusion matrices for cross-target gesture recognition on the DUAL-Net and MM-Fi datasets. (**a**) Results on the DUAL-Net dataset. (**b**) Results on the MM-Fi dataset.

**(2) Confusion matrix-based analysis:** To further evaluate cross-target recognition behavior, we conduct a per-class analysis using the confusion matrix under target-user adaptation. In this experiment, the model is first trained on source-domain data and then adapted to the target user using CMG-generated WiFi samples. The corresponding results on the DUAL-Net and MM-Fi datasets are shown in Figure 20, where (a) and (b) represent the two datasets, respectively. As shown in Figure 20a, most categories in the DUAL-Net dataset are correctly classified, with strong diagonal dominance. The main confusion occurs between G6 and G1, where G6 (*draw zigzag*) involves rapid multi-directional arm movements that partially overlap with the forward extension pattern of G1 (*push–pull*), leading to similar CSI temporal variations. A similar pattern is observed in Figure 20b. The primary misclassification occurs between G2 (*vertical arm stretch*) and G4 (*right arm extension*). Both gestures involve prominent single-arm raising or extension movements, which induce comparable amplitude fluctuations and similar energy distribution patterns in CSI signals, making them difficult to distinguish under target-user variations. Overall, the confusion matrices indicate that remaining errors mainly arise from gestures with similar motion structures and correlated CSI response patterns. While the proposed method effectively captures most class distinctions, it may still be limited in distinguishing gestures with highly similar motion trajectories and overlapping CSI variations.

**(3) Complexity and runtime analysis.** DUAL-Net follows a two-stage learning paradigm consisting of offline representation learning and online user adaptation. The contrastive fusion module and the SMD-guided cross-modal generation module are employed only during offline training, which can be performed on cloud or server platforms. During deployment, gesture recognition relies on WiFi measurements and the trained recognition network. The visual-guided adaptation and diffusion-based feature generation processes are confined to the offline learning stage and are not required during online inference. Consequently, the additional computational cost introduced by these components does not affect deployment efficiency.

To quantify the computational overhead of the online stage, we evaluate the complexity and runtime of DUAL-Net under the target-user adaptation setting. The results are averaged over three profiling trials on CUDA with a batch size of 64. As shown in Table 4, DUAL-Net contains 107.40 M parameters, among which 57.93 M are trainable. The feature extraction model contains 49.75 M parameters, while only 0.28 M parameters are updated during adaptation. The classifier contains 57.65 M trainable parameters and serves as the primary component for decision boundary adaptation. The inference latency of DUAL-Net is 102.10 ms per batch, corresponding to 1.60 ms per sample and a throughput of 626.84 samples per second.

## 6. Conclusions

This paper presents DUAL-Net, a domain-invariant and user-adaptive learning framework for WiFi-based gesture recognition. The core of the method lies in a contrastive fusion learning (CFL) module, which learns domain-invariant representations by aligning complementary information from WiFi and vision modalities, and a spatial matrix difference (SMD)-guided cross-modal generation (CMG) module, which generates user-adaptive WiFi features conditioned on structural cues to capture inter-user variations. Extensive experiments on the MM-Fi dataset and a self-collected dataset demonstrate that DUAL-Net consistently improves cross-user recognition performance compared with existing methods, highlighting the effectiveness of jointly modeling domain-invariant and user-adaptive representations. Overall, these results indicate that combining CFL-based cross-modal representation learning with SMD-guided CMG is an effective strategy for reliable WiFi-based gesture recognition. In addition, the proposed two-stage learning framework enables efficient deployment by separating offline multimodal learning from online inference. In future work, we will extend the framework to more complex multi-user and multi-scene settings while addressing deployment challenges such as computational overhead, latency, and scalability. We will also investigate robustness under missing modalities and explore additional sensing modalities such as RFID and mmWave to improve generalization.

## Figures and Tables

**Table 1:** WiFi encoder configuration.

ID	Operation	Configuration
Input	CSI amplitude	DUAL-Net: 1×200×270
		MM-Fi: 1×200×342
1	Conv2d	Kernel = 2×2, FM = 64
	MaxPooling2d	Stride = 3
	BatchNorm2d	FM = 64, ReLU
2	Conv2d	Kernel = 2×2, FM = 64
	MaxPooling2d	Stride = 1
	BatchNorm2d	FM = 64, ReLU
3	Conv2d	Kernel = 1×1, FM = 32
	BatchNorm2d	FM = 32, ReLU
4	Conv2d	Kernel = 1×1, FM = 16
	BatchNorm2d	FM = 16, ReLU

**Table 2:** Vision encoder configuration.

ID	Operation	Configuration
Input	RGB video clip	1×10×160×212
		10 sampled frames
1	Conv3d	Kernel = 2×5×5, FM = 64
	MaxPooling3d	Stride = 3
	BatchNorm3d	FM = 64, ReLU
2	Conv3d	Kernel = 2×5×5, FM = 64
	MaxPooling3d	Stride = 3
	BatchNorm3d	FM = 64, ReLU
3	Conv3d	Kernel = 1×5×5, FM = 32
	BatchNorm3d	FM = 32, ReLU
4	Conv3d	Kernel = 1×2×5, FM = 16
	BatchNorm3d	FM = 16, ReLU

**Table 3:** Summary of the datasets used for evaluation (# denotes the number of items).

Dataset Name	Modality	# Gestures	# Volunteers	# Envs	Env. IDs	# Samples
DUAL-Net	WiFi, RGB	6	10	5	A, B, C, D, E	1800
MM-Fi [54]	WiFi, RGB, Depth, LiDAR, mmWave	9	10	4	E01, E02, E03, E04	8640

**Table 4:** Complexity and runtime statistics of DUAL-Net under the target-user adaptation setting.

Metric	Value
Total parameters	107.40 M
Trainable parameters	57.93 M
Feature extraction model parameters	49.75 M
Trainable feature extraction model parameters	0.28 M
Classifier parameters	57.65 M
Batch size	64
Latency	102.10 ms/batch
Per-sample latency	1.60 ms/sample
FPS	626.84 samples/s

## Data Availability

The data presented in this study are available upon request from the corresponding authors.
